# Effects of poling and crystallinity on the dielectric properties of Pb(In_1/2_Nb_1/2_)O_3_-Pb(Mg_1/3_Nb_2/3_)O_3_-PbTiO_3_ at cryogenic temperatures

**DOI:** 10.1038/s41598-019-38995-9

**Published:** 2019-02-21

**Authors:** Philippa M. Shepley, Laura A. Stoica, Yang Li, Gavin Burnell, Andrew J. Bell

**Affiliations:** 1 School of Chemical and Process Engineering, University of Leeds, Leeds, UK; 2 School of Physics and Astronomy, University of Leeds, Leeds, UK

## Abstract

The mechanisms underlying the anomalously large, room temperature piezoelectric activity of relaxor-PbTiO_3_ type single crystals have previously been linked to low temperature relaxations in the piezoelectric and dielectric properties. We investigate the properties of Pb(In_1/2_Nb_1/2_)O_3_-Pb(Mg_1/3_Nb_2/3_)O_3_-PbTiO_3_ between 10 and 300 K using dielectric permittivity measurements. We compare results on single crystal plates measured in the [001] and [111] directions with a polycrystalline ceramic of the same composition. Poled crystals have very different behaviour to unpoled crystals, whereas the dielectric spectrum of the polycrystalline ceramic changes very little on poling. A large, frequency dependent dielectric relaxation is seen in the poled [001] crystal around 100 K. The relaxation is much less prominent in the [111] cut crystal, and is not present in the polycrystalline ceramic. The unique presence of the large relaxation in poled, [001] oriented crystals indicates that the phenomenon is not due their relaxor nature alone. We propose that heterophase dynamics such as the motion of phase domain boundaries are responsible for both the anomalous electromechanical and dielectric behaviour.

## Introduction

Single crystal relaxor-PbTiO_3_ ferroelectric materials can have exceptionally high piezoelectric properties at room temperature. Their large piezoelectric and dielectric constants, along with low dielectric losses are desirable for a wide range of applications^1–3^. Understanding the physical mechanisms responsible for the properties of single crystal relaxor-PbTiO_3_ ferroelectric materials can provide useful insights for the development of new piezoelectric materials. Much of the recent effort to understand the origins of the excellent room temperature properties of relaxor-PbTiO_3_ materials has focused on understanding the piezo- or dielectric behaviour of the materials below room temperature. Relaxation step features in piezoelectric and dielectric properties at low temperatures have been reported in single crystal relaxor-PbTiO_3_ samples^4–6^, in addition to the characteristic relaxor-ferroelectric dielectric peaks above room temperature^6–9^. It has been suggested that the low temperature relaxations in relaxor-PbTiO_3_ crystals are related to mechanisms that produce the room temperature properties^6^.

For rhombohedral Pb(Mg_1/3_Nb_2/3_)O_3_-PbTiO_3_ (PMN-PT) crystals, Martin *et al*. and Li *et al*.^4,10^ have shown that at around 200 K the reduction in dielectric permittivity and piezoelectricity with temperature levels off, before dropping sharply between 100 K and 20 K. The drop in permittivity is associated with a peak in the dielectric loss, both of which show a large variation as a function of driving frequency. The step feature suggests a “freezing out” of temperature activated dynamics, which has been cited as evidence that the persistence of polar nano-regions down to lower temperatures gives relaxor-PbTiO_3_ materials their high room temperature properties^6,11^.

To explore the implications of low temperature relaxations in dielectric data we can compare data available for a range of materials. Low temperature dielectric data from different ferroelectric and relaxor-ferroelectric materials show a wide range of anomalies and features at cryogenic temperatures. The large dielectric relaxation feature highlighted by Li *et al*.^6^ is not always present in relaxor-PbTiO_3_ single crystals^7,12,13^. Studies on PMN-PT suggest that the material composition and poling state^7^ influence the size, shape and presence of a low temperature feature. Work on PMN-PT ceramics^14,15^ showed broad dielectric loss anomalies that appear more similar to some data on lead zirconate titanate (PZT) based ceramics than relaxor-PbTiO_3_ single crystals^16–18^. In PZT ceramics, freezing out of the motion of domain walls is used to explain broad peaks in the dielectric loss spectra^10^. There are dielectric data on Fe doped PZT ceramics^19^ showing a step feature with frequency dispersion very similar to that seen in PMN-PT^4,6^. Arlt *et al*. found good agreement between this data and their domain wall dynamics model^19^.

The low temperature, rhombohedral phase of BaTiO_3_ can give dielectric data that peak to anomalously high values, then reduce close to 0 K^20–23^. The peaks are similar to those seen in PMN-PT in their shape and frequency dispersion. The presence of this low temperature relaxation behaviour in ferroelectric BaTiO_3_ single crystals has been shown to vary between crystals grown by different methods, and depending on the crystals’ electric field and temperature histories^20,22^. Wang *et al*. have shown that images of different domain states can be linked to differences in the size of the dielectric constant peak in the rhombohedral phase of BaTiO_3_ single crystals^23^.

In order to understand the origins of low temperature anomalies and their relationship to room temperature properties, data on materials in a range of conditions are required. Here we investigate the relaxation step in relaxor-PbTiO_3_ materials by considering polycrystalline ceramic and single crystal Pb(In_1/2_Nb_1/2_)O_3_-Pb(Mg_1/3_Nb_2/3_)O_3_-PbTiO_3_ (PIN-PMN-PT). We compare poled (001) and (111) cut crystals to explore the effect of an ordered domain structure with many domain walls and the effect of a state where there are nominally no domain walls. We investigate the effects of poling the material and find that the large relaxation step only becomes apparent when the single crystal is poled.

## Results

**Figure 1:**
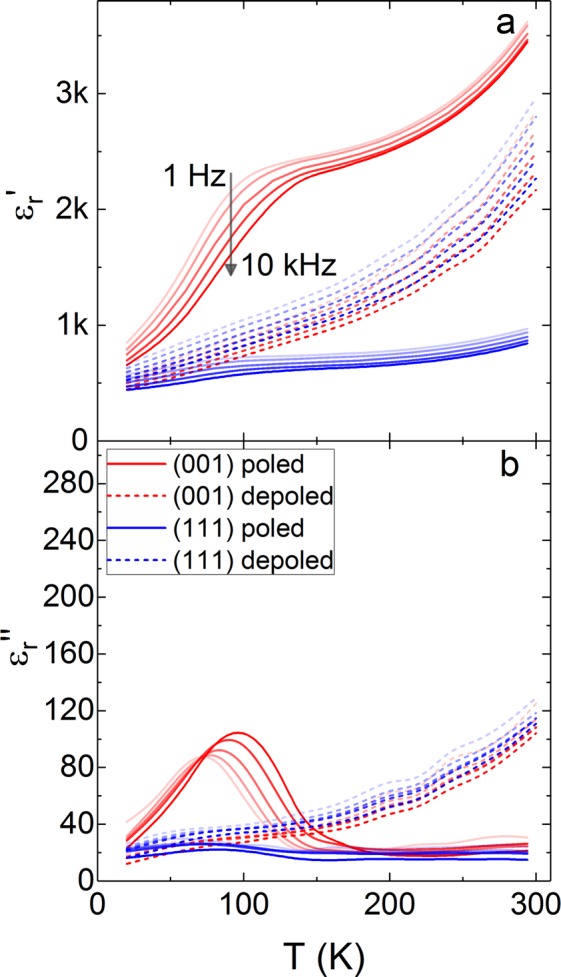
The (**a**) real and (**b**) imaginary parts of the dielectric permittivity in (001) and (111) cut PIN-PMN-PT single crystals are shown at temperatures from 20 K to 300 K. The solid, red lines are for the poled (001) cut and the dashed, red lines are for the depoled (001) cut. The solid, blue lines are for the poled (111) cut and the dashed, blue lines for the depoled (111) cut.

We have studied relaxor-ferroelectric PIN-PMN-PT below room temperature. The nominal composition of the material, PIN_0.28_-PMN_0.40_-PT_0.32_, was chosen to be close to the morphotropic phase boundary (MPB) and give a rhombohedral structure at room temperature. The data are from PIN_0.28_-PMN_0.40_-PT_0.32_ crystal plates cut with (001) and (111) faces, and from a polycrystalline ceramic pellet. Details of sample preparation and structural characterisation are available elsewhere^24^. The PIN-PMN-PT crystals have a rhombohedral-tetragonal transition at 420 K and depole at 440 K. The polycrystalline material has a rhombohedral-tetragonal transition at 410 K and depoles at 475 K. The (001) cut crystal has a room temperature d_33_ of 1150 ± 20 pC/N, the (111) cut crystal has a room temperature d_33_ of 75 ± 1 pC/N and the d_33_ of the polycrystalline material at room temperature is 163 ± 3 pC/N. The real and imaginary parts of the dielectric permittivity, ${\varepsilon ^{\prime} }_{{\rm{r}}}$ and ${\varepsilon ^{\prime\prime} }_{{\rm{r}}}$, measured for the (001) and (111) cut PIN-PMN-PT crystals, in a poled and depoled state, are plotted in Fig. 1. We see the same features in the dielectric properties of (001) poled rhombohedral PIN-PMN-PT as have been reported for PMN-PT^6^, however, we find differences between the two crystal cuts and large differences between the depoled and poled crystals.

The real part of the permittivity ${\varepsilon ^{\prime} }_{{\rm{r}}}$ of the (001) cut crystal increases when the sample is poled, whereas for the (111) cut ${\varepsilon ^{\prime} }_{{\rm{r}}}$ decreased after poling. For both crystals, the frequency dispersion at room temperature is reduced by poling, however the room temperature values of permittivity are very different.

The poled (001) crystal has the permittivity step feature seen in PMN-PT, where the rate of decrease of ${\varepsilon ^{\prime} }_{{\rm{r}}}$ as the sample is cooled slows at 200 K, then increases around 100 K, so that ${\varepsilon ^{\prime} }_{{\rm{r}}}$ drops sharply. The feature is also present to some degree in the (111) crystal, although the size and sharpness of the drop is much less significant than in the (001) crystal.

The imaginary part of the permittivity ${\varepsilon ^{\prime\prime} }_{{\rm{r}}}$ is low at room temperature in the poled crystals. There is very little change in ${\varepsilon ^{\prime\prime} }_{{\rm{r}}}$ from the room temperature value in the (111) crystal. In the (001) crystal the ${\varepsilon ^{\prime} }_{{\rm{r}}}$ step feature is associated with a large peak in ${\varepsilon ^{\prime\prime} }_{{\rm{r}}}$.

The behaviour with temperature of the two depoled PIN-PMN-PT crystals, (001) and (111) cut, is almost identical. There is a large variation in the relative permittivity ${\varepsilon ^{\prime} }_{{\rm{r}}}$ of depoled PIN-PMN-PT with driving frequency. The frequency dispersion is largest at room temperature, then below 150 K the frequency dispersion begins to decrease. The imaginary part of the permittivity ${\varepsilon ^{\prime\prime} }_{{\rm{r}}}$ follows a similar function of temperature as ${\varepsilon ^{\prime} }_{{\rm{r}}}$, dropping to approximately 20% of its room temperature value by 20 K, with no prominent step features.

**Figure 2:**
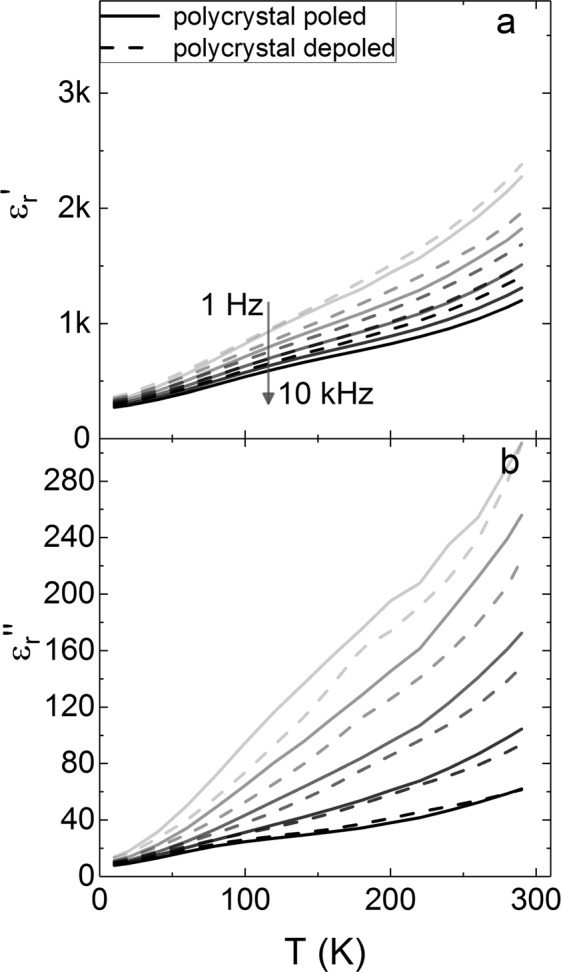
The (**a**) real and (**b**) imaginary parts of the dielectric permittivity in a PIN-PMN-PT polycrystalline ceramic are shown at temperatures from 10 K to 300 K. The solid lines are for the poled ceramic and the dashed lines are for the depoled ceramic.

The polycrystal behaves in a similar way to the depoled crystals. The real and imaginary parts of the dielectric permittivity, ${\varepsilon ^{\prime} }_{{\rm{r}}}$ and ${\varepsilon ^{\prime\prime} }_{{\rm{r}}}$, were measured for the ceramic in a poled and depoled state and the results are shown in Fig. 2. There is a substantial difference as a function of frequency in the dielectric data (both real and imaginary parts), which closes as the temperature approaches 0 K. The slopes of relative permittivity ${\varepsilon ^{\prime} }_{{\rm{r}}}$ and the imaginary part of the permittivity ${\varepsilon ^{\prime\prime} }_{{\rm{r}}}$ are almost constant over the measured temperature range, although the data at 1 Hz are considerably steeper than the data at 10 kHz. In the range of temperature and frequency in Fig. 2 there is very little change in the permittivity spectra of the polycrystal when it is poled compared to when it is depoled.

**Figure 3:**
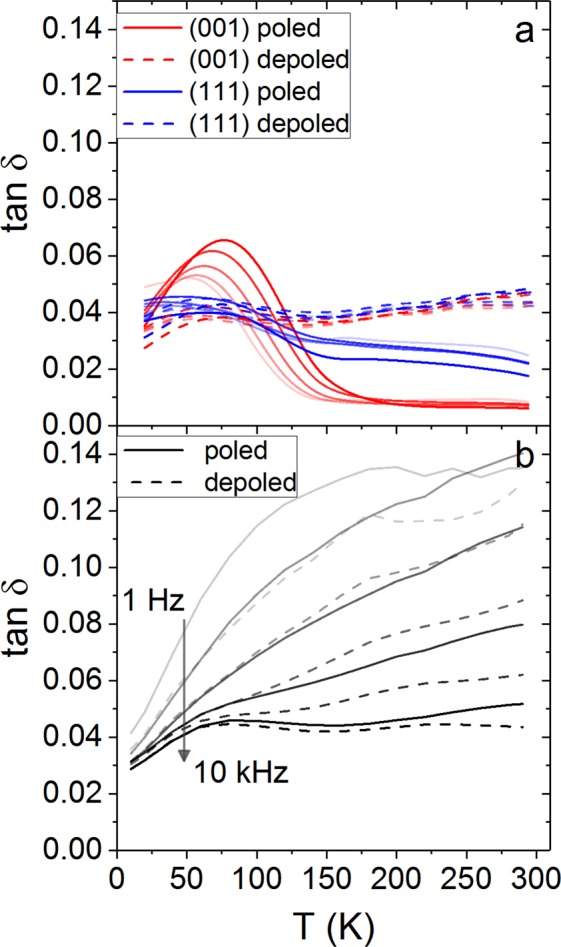
The dielectric loss in (**a**) (001) and (111) cut PIN-PMN-PT single crystals and (**b**) a polycrystalline ceramic are shown at temperatures from 10 K to 300 K. The solid lines are for the poled material and the dashed are for the depoled material. The red lines are for the (001) cut, the blue lines are for the (111) cut and the black lines are for the polycrystalline ceramic.

In addition to the real and imaginary parts of the dielectric permittivity, we also show the dielectric loss tangent for all samples in Fig. 3. For the single crystal samples the low temperature features in the imaginary permittivity (Fig. 1b) and the dielectric loss (Fig. 3a) are qualitatively similar. The peak in the (001) data represents a maximum in the energy lost when changing the polarisation direction.

For the polycrystalline ceramic the dielectric loss in Fig. 3b shows a more prominent low temperature effect than the permittivity in Fig. 2. The dielectric loss in the polycrystal changes less than ${\varepsilon ^{\prime\prime} }_{{\rm{r}}}$ close to room temperature, and we then see larger changes at lower temperatures. Loss data at frequencies below 1 kHz reduces more steeply below 150 K, whereas for higher frequency data there is small bump that could indicate a high frequency process that freezes out below 100 K.

**Figure 4:**
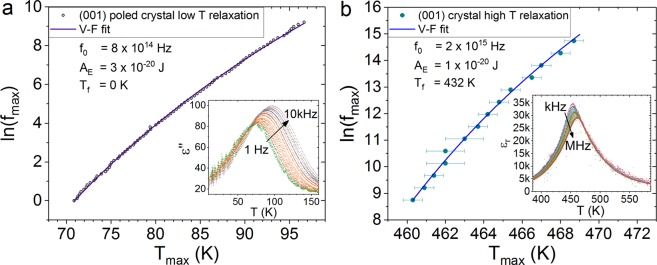
The relaxation peak data are plotted as the natural logarithm of the frequency and the temperature of the peak maxima. The points are the peak maxima position data and the lines are least squares fits to Equation 1. (**a**) Shows the low temperature imaginary permittivity relaxation data for the poled (001) cut crystal and (**b**) shows the high temperature relative permittivity peak data for the (001) cut crystal during cooling from the paraelectric state. The insets show the data used to extract the peak positions.

To investigate the low temperature relaxation mechanism we have extracted the frequencies and temperatures of the imaginary permittivity relaxation peaks for the (001) cut crystal (Fig. 4a). A common approach for analysing high temperature dielectric relaxation peaks in relaxor materials is to fit the Vogel-Fulcher equation^25–28^ for the peak frequencies given by

1 $${f}_{max}={f}_{0}\,\exp [{A}_{E}/({T}_{max}-{T}_{f})],$$

with peak temperatures *T*_max_, to extract a scaling frequency *f*_0_, a freezing temperature *T*_*f*_ and an activation energy *A*_*E*_. We have fitted the low temperature (001) cut crystal peak data in Fig. 4a. The fitted freezing temperature is 0 K, meaning that the Vogel-Fulcher has reduced to an Arrhenius law with no freezing temperature. For comparison, we show the same analysis for the high temperature permittivity data from the same (001) cut crystal in Fig. 1b. In the case of the high temperature permittivity data, the Vogel-Fulcher fit gives a freezing temperature of 432 K. The high temperature relaxation is consistent with glass-like freezing dynamics, whereas the low temperature relaxation is not.

## Discussion

Low temperature dielectric relaxation data have been modelled and explained by freezing-out of dynamics associated with both domains^19,23^ and polar nano-regions^11^. The preparation conditions of materials, including the crystal growth method, crystalline or polycrystalline nature and the field and temperature histories are all important factors in the low temperature dielectric response.

The relaxation step that we measure in the PIN-PMN-PT (001) single crystal is very similar to both the step seen in PMN-PT^6,11^ (which has been modelled by dynamics of polar nano-regions) and in rhombohedral BaTiO_3_^22,23^ (which has been modelled by dynamics of domain walls). Comparing the dielectric permittivity versus temperature of single crystals and a polycrystal in poled and depoled states plotted in Figs 1 and 2 shows that the low temperature step feature is only present in poled single crystals. The difference in the low temperature dielectric properties between single crystal and polycrystalline material with the same composition shows that the relaxation features reported here and by other researchers may not be entirely a consequence of the relaxor-like behaviour of polar nano-regions in a ferroelectric matrix, or of motion of ferroelectric domain walls.

In unpoled crystals and ceramics, domains representing all crystallographically allowed polarisation directions can be present. Applying a small measurement field favours some of these domains, allowing them to expand at the expense of others. Small changes to the domain configuration give rise to an excess change in polarisation compared to the lattice response. This phenomenon, known as the domain wall contribution, appears in both the permittivity and piezoelectric charge coefficient.

Poling an as-grown crystal changes the polarisation configuration so that only domains with a polarisation component parallel to the applied field remain. For a rhombohedral PIN-PMN-PT crystal poled along [111], the polarisation is aligned to [111] only, giving a nominally single domain configuration. Hence, the domain wall response is eliminated or reduced compared to the unpoled crystal, as is observed in Fig. 1 where there is a significant reduction in permittivity in the [111] poled crystal.

Poling the ceramic also results in a decrease in permittivity (see Fig. 2a), although the effect is small compared to the (111) cut crystal. The random orientation of crystal grains in the ceramic with respect to the applied field results in only partial elimination of domain walls, leaving a large population of domain walls between inequivalent domain states. Hence there is still a substantial domain wall contribution in the poled ceramic.

In contrast, poling an (001) cut crystal results in a large increase in the permittivity, which reduces dramatically below 100 K (Fig. 1a). For a [001]-poled rhombohedral PIN-PMN-PT crystal, we expect four domain variants with polarisation parallel to each of the four 〈111〉 axes that have a positive [001] component. According to the conventional model^1^, the four domains are degenerate with respect to applied fields along [001], so there should be no permittivity contributions from domain wall motion, although contributions from static domain walls are possible^29,30^. Rayleigh studies on PIN-PMN-PT have shown that the extrinsic contributions from domain walls account for as little as 2% of the piezoelectric effect in (001) cut single crystals^31^. Hence, we cannot attribute the increased permittivity and its low temperature relaxation to conventional domain wall translation. According to the conventional model^1^, the enhanced permittivity is due to polarisation rotating towards and away from the [001] direction under applied fields. However, such a mechanism, in which the polarisation rotates within domains, is not temperature activated, so the observed low temperature relaxation is not consistent with this model.

Other evidence suggests that for PIN-PMN-PT^8^ and PMN-PT^32–34^ crystals with compositions close to the MPB, poling changes the crystallographic symmetry. X-ray diffraction shows that field cooling a crystal that is initially rhombohedral at room temperature, produces a monoclinic symmetry. A difference in crystal symmetry between the poled and depoled samples would change the available domain states. However, the resulting fully-poled domain structure should still be invariant to applied fields along [001] and hence domain wall translation contributions to permittivity would only result if poling were incomplete or “back-switching” occurred. As there is no evidence that the low temperature relaxation in [001]-poled crystals is dependent upon the magnitude of the poling field, we suggest that such a mechanism does not make a substantial contribution.

There is also evidence that the apparent monoclinic structures in materials close to the MPB may be due to the averaging of variations in local structure, such as a combination of rhombohedral and tetragonal nano-domains^35,36^. Poling relaxor-PbTiO_3_ materials close to the MPB may therefore enhance nanoscale structural variations that emerge from compositional variations, giving rise to phase domains with different crystal symmetries and polarisation orientations. Theoretical work shows that rhombohedral and tetragonal regions can coexist in PMN-PT^37^ and there have been experimental observations of tetragonal nanodomains in PMN-PT^38,39^. In the case of both PMN-PT and PIN-PMN-PT, the monoclinic symmetry is one in which the average polarisation vector is close to [111], hence if this were a composite structure of tetragonal and rhombohedral nanodomains, the tetragonal phase would be a relatively small volume fraction.

Phase domain configurations could comprise either static or dynamic components, or some combination of both. In the case of a static tetragonal component in a rhombohedral matrix, applying a field parallel to [001] would result in growth of the tetragonal volume fraction. As the polarisation along [001] in the tetragonal phase is larger than the [001] component in the rhombohedral phase, this mechanism would significantly enhance the permittivity and piezoelectric activity, giving the enhanced properties seen in relaxor-PT crystals. Domain walls can have a different symmetry to the bulk, so may act as nucleation centres for phase domains. Rao and Wang^40^ have simulated the behaviour of ferroelectric domain walls under applied fields and found that domain wall broadening contributes to the macroscale properties. In an (001) poled rhombohedral crystal, domain walls with tetragonal symmetry broaden under applied fields, whereas in a depoled crystal the effect is balanced by narrowing of domain walls with opposite polarisation at their cores. In the dynamic case, in which tetragonal fluctuations appear in the rhombohedral matrix, an [001] applied field would stabilize and increase the tetragonal volume fraction, with similar effect. For an energy landscape in which there is a barrier between the tetragonal and rhombohedral energy minima, both mechanisms would be temperature activated and consistent with the observed low temperature relaxation of the permittivity.

## Conclusions

We have presented dielectric data below room temperature for the relaxor-ferroelectric material PIN-PMN-PT. We compare PIN-PMN-PT measured in six different conditions: poled and depoled single crystal (001) cut, poled and depoled single crystal (111) cut, and poled and depoled polycrystalline ceramic.

The large dielectric relaxation feature reported in relaxor-PbTiO_3_ materials is only present in the poled single crystals, and is much more prominent in the multi-domain (001) cut than the single-domain (111). We find that in contrast to the high temperature dielectric relaxation peaks, the positions of the low temperature relaxation peaks in the (001) cut crystal fit an Arrhenius rather than a Vogel-Fulcher function, showing that the mechanism does not involve glass-like freezing dynamics.

The differences between sample material under different conditions show that low temperature relaxations in relaxor-PbTiO_3_ materials cannot be fully explained by macroscopic polarisation rotation^1^, by the dynamics of polar nano-regions in a ferroelectric matrix^6,11^ or by the conventional translation of ferroelectric domain walls^19^. The polar nano-region model would suggest that the relaxation should be present in all of the PIN-PMN-PT samples and would lead us to expected to find a non-zero freezing temperature from the Vogel-Fulcher fit to the low temperature relaxation peak data. The domain wall motion model would suggest that the relaxation should be present in all the unpoled samples, but not in the single domain (111) crystal. Since we have shown that these mechanisms don’t account for all of our data, we suggest other temperature activated candidates: the dynamic fluctuations of nanoscale domains between rhombohedral and tetragonal phases, or the motion of phase domain walls. In the case where domain walls have a different structure to domains, the motion of phase boundaries may be linked to broadening of domain walls under applied fields.

## Methods

We have studied crystal plates cut with (001) and (111) faces, and a polycrystalline ceramic pellet. To make the samples, powdered material was prepared by mixed oxide methods. The polycrystalline ceramic pellet was formed by sintering and the crystal was grown by Bridgman technique^24^. Silver epoxy was painted onto the crystal and pellet main faces and cured at 770 K to form electrodes. The (001) cut crystal was 1.117 mm thick and had an electrode area of 0.2019 cm^2^, the (111) cut crystal was 1.200 mm thick and had an electrode area of 0.2601 cm^2^, and the ceramic pellet was 1.720 mm thick and had an electrode area of 0.7557 cm^2^. The nominal composition of all of the material is PIN_0.28_-PMN_0.40_-PT_0.32_, which is close to the morphotropic phase boundary (MPB) and gives a rhombohedral structure at room temperature^24^.

The samples were all prepared for measurements in the poled state by first annealing to 850 K, a point well above any phase transitions or dielectric maxima, then allowing them to cool to room temperature. We poled the samples by heating them to 370 K, then applying an electric field of 1 kV/mm while the samples cooled to room temperature. The elevated temperature is high enough to lower the energy barrier for domain re-orientation, but is below any phase transitions. All samples showed piezoelectric resonance peaks at high frequency, indicating that they were properly poled. The measurements in a depoled state were taken after the samples had been annealed to a point (above 500 K) where they no longer showed a spontaneous polarisation or piezoelectric resonance peaks.

The real and imaginary parts of the dielectric permittivity, ${\varepsilon ^{\prime} }_{{\rm{r}}}$ and ${\varepsilon ^{\prime\prime} }_{{\rm{r}}}$, were measured with a Solartron impedance analyser and XM-Studio MTS software. The crystals were mounted in an Oxford Microstat, where the temperature was swept at a rate of 2 K/minute between 10 K and 300 K. A driving voltage with an rms value of 2 V was applied at a range of frequencies between 10 kHz and 0.05 Hz, and the response was measured. The high temperature measurements for Vogel-Fulcher analysis were taken with a HP4192A impedance analyser while cooling from 800 K in a tube furnace.

The rhombohedral-tetragonal transition temperature and depoling temperature were determined by both permittivity measurements in the tube furnace and by polarisation versus temperature measurements in the cryostat. The room temperature d_33_ measurements were taken with a Berlincourt meter.

## Data Availability

The full set of data is available from 10.5518/407.
